# Self-tracking the microbiome: where do we go from here?

**DOI:** 10.1186/s40168-015-0138-x

**Published:** 2015-12-12

**Authors:** Carine Gimbert, François-Joseph Lapointe

**Affiliations:** Département de sciences biologiques, Université de Montréal, CP. 6128, Succursale Centre-ville, Montréal, QC H3C 3J7 Canada

**Keywords:** Microbiome, Quantified self, Self-tracking, Self-experimentation, Participant-led research, Personalized medicine, Citizen science

## Abstract

The quantified self community brings together enthusiasts who are using technological devices to monitor their health and social media to share their personal data with others online. In light of the growing popularity of this movement, self-trackers are challenging the health-care system by raising important questions about data ownership and risk-taking. As we enter a new era of consumer genomics, a significant number of quantified self (QS) individuals are now interested in the monitoring of their microbiome and performing personal interventions. In this paper, we discuss the scientific validity of experiments involving serial observations of a single individual as opposed to randomized clinical trials. We look at self-tracking from an ethical standpoint by questioning the risks and assessing the potential benefits for personalized medicine in general and for microbiome research in particular.

## Background

With the advent and recent acceptance of self-tracking, a large number of individuals are now relying on technological tools and wearable sensors to monitor and analyze their daily activities as a way of improving performance, productivity, or any other factors involved in personal well-being [1]. Nowhere is this so-called quantified self (QS) movement more active and important than in medicine [2–4], with online blogs [5, 6], and self-help books [7] of people claiming to have cured specific diseases on their own using self-tracking and data logging. With annual conferences, workshops, online forums, and specialized journals, this large community of people primarily motivated by health issues is challenging the medical profession and pushing for a change.

Although it is currently impossible to monitor the microbiome at home, some crowdfunded biotech start-ups [8, 9] are offering a paying service to analyze one’s microbiome as part of a larger citizen science movement [10]. Using data visualization tools, one could then find out what types of people have a microbiome like theirs and understand how their microbiome changes over time. With this also comes the possibility of laypersons doing experiments and personal interventions on their microbiota to alleviate a specific disease or health condition [11]. Sure enough, individualized microbiome analyses will be of growing interest in the years to come [12]. In this paper, we will discuss the pros and cons of personal experimentations, performed either by amateurs or scientists, from a scientific and ethical standpoint, questioning the shortcomings and potential benefits of self-tracking for microbiome research.

## Main text

### What is self-tracking?

Self-tracking involves collecting all sorts of data which can be measured about the self, including biological, psychological, physical, behavioral, or environmental information [1], as a way to optimize a specific lifestyle and resolve a personal problem or disease, such as sleep disorder, mood swings, sexual dysfunction, diabetes, obesity, and irritable bowel syndrome. To do so, self-trackers are using digital tools, wearable devices, connected gadgets, and computer apps to collect, monitor, analyze, visualize, interpret, and share their data online with the general public and other members of the community [13].

The jury is still out about the real benefits (efficiency/applicability) of self-tracking in medicine, despite its increasing popularity [14, 15]. On the other hand, self-experimentation has a long tradition in medical research, with several breakthrough discoveries attributed directly to this mode of private practice [16]. Here, we posit that self-trackers are a new breed of self-experimenters, whether amateur or professionals, a category of individuals motivated by their own health, who are conducting experimental studies on themselves. In self-tracking, the user, practitioner, and the beneficiary of the study are the same person. In self-experimentation, the practitioner is the user, while the beneficiaries can be anyone else. Yet, the process of measuring, analyzing, and understanding one’s *personal data* is slowly being replaced by *group data* collected by individual self-trackers driven by similar goals and using standardized protocols to serve the community as a whole [17]. This methodological shift from *private* self-tracking to the *pushed*, *communal*, *imposed*, and *exploited* types of self-tracking [18] has important implications. Namely, as more and more of these community-based projects will be undertaken, an exponential amount of information will be made available, raising concerns about data ownership [19], integration, and quality.

QS individuals indirectly contributed to the emergence of biocitizenship [20], a movement dedicated to empower individuals to take care of their own health [21, 22]*.* Self-trackers are also part of the larger field of digitally engaged patients [23] advocating self-monitoring and self-care as an alternative approach to medicine. As such, they represent a driving force for the promotion of predictive, preventive, personalized, and participatory (P4) medicine, as envisioned by Leroy Hood to transform the existing health-care system [24, 25]. In spite of its promising future, serious concerns have been raised about the scientific robustness and ethical validity of quantified self-tracking, either performed by a single individual or by a group of individuals.

### Is self-tracking scientifically valid?

As with any study based on a single individual, self-tracking suffers from severe shortcomings: scientific validity and generalization. Besides these obvious flaws, objections to self-research also question the difficulty of controlling for environmental and hereditary variables, lack of models to help in the conduct of self-experimentation, and the well-know placebo effect [1]. Because self-tracking studies are not randomized or blinded in traditional ways, also comes the problem of publication in peer-reviewed journals. But above all, it is the personal involvement and motivations of the experimenter, which for some, make the research less objective and more open to questionable interpretation.

Modern self-trackers are not unaware of the problems associated with studies based on a single subject, and they are constantly trying to find new ways, individually or collectively, to improve their methods and further scientific acceptance of their results. The fact is that many persons involved in self-tracking are *bona fide* researchers, with a background in experimental science. Some are actually using grant money and lab resources to perform long-term vertical studies [26], while others are doing this as a hobby, outside of their main research area [27], even claiming that, in specific cases, self-experimentation was far better than their mainstream research [28].

Advocates and practitioners of self-tracking and self-experimentation argue that advantages of individual-based studies are numerous. For one, it is far cheaper than conventional research using clinical trials. It is also easier to do and more flexible, thus allowing to study a wider range of problems by measuring many things at once [28]. Additional benefits of self-tracking are about velocity of question asking and the multiplicity of experiment iterating [1]. As the research questions are prompted by the users in a bottom-up fashion, data can be gathered much more rapidly, thus accelerating the whole experimental process. In the spirit of citizen science, self-tracking data are available online for everybody to use, which engages the whole community to take part in the process of scientific discovery, either by combining data, comparing data or re-analyzing the data of a particular experiment [3]. And by combining personal data with data collected from a group of participants motivated by a similar problem, the information begins to resemble more traditional scientific experiments and clinical trials [29].

In spite of this, the fundamental question remains. In the absence of standardized research protocols and analytical procedures for studying private behavior, can the credibility of self-experimentation as science consist in the generality and replicability of the method [30], or in other words, can there be a science of *n* = 1? In the context of microbiome research, where *n* = me, this question takes a whole different meaning, since “me” is more than just human cells. Self-tracking the microbiome is not about tracking single individuals, it is about studying an entire ecosystem of microbes representing *N* different species and *M* different genes. Typically, as *N* and *M* are respectively in the hundreds and thousands, someone interested in the monitoring of the gut microbiome can collect a massive amount of metagenomic data, large enough to test specific hypotheses with great statistical significance, even more so, if one were also to collect data from other body sites, such as the vaginal microbiome, the oral microbiome, or the skin microbiome.

### Is self-tracking ethically acceptable?

In addition to its scientific soundness, the ethical validity of self-experimentation has been debated at length [31]. Sure enough, self-tracking is convenient: getting informed consent is a given, requiring neither conversation with the subjects nor complex paperwork; the subject/investigator is well informed and understands the research protocol, compliance is not a problem, and cash inducements are not required [32]. Taken at face value, the moral advantages of self-experimentation are many, with maximum identification of the subjects, understanding, and spontaneity. However, these theoretical advantages are not enough to make self-experimentation ethically acceptable [33]. For one, people who self-experiment with devices, get testing that is available direct-to-consumer or participate in crowdfunded research all have to pay for these devices and tests. This creates a gap between the people who can afford to do this type of self-experimentation and people who cannot afford it. There is then the potential to exacerbate existing health disparities. In addition, the analysis of one’s personal data by a third party also raises the problem of dual-use research and innovation [34] and its precautionary aspects, namely, when data sharing can lead to both beneficial and harmful uses [35].

Of course, no one can stop individuals to perform any type of self-experiments on themselves, even dangerous ones. It is when other participants are recruited for group self-experimentations and other *exploited* types of self-tracking [18] that ethical problems may arise. Davis [32] proposes a framework for evaluation of such projects using three criteria: the good faith argument, the golden rule argument, and the risk argument. To the extent that self-experimentation is neither necessary nor sufficient to justify enrolling non-investigators, he concludes that there is no ethical reason to encourage group self-experimentation [32]. Similarly, there is a significant gap in ethical oversight with regard to bottom-up, participant-led health research [36]. According to some, Institutional Review Boards (IRBs) are structured for the old world of scientific inquiry, whereas citizen science blurs the lines between the research subject and the researchers [37]; in self-tracking, the two are the same person. One interesting proposal yet to be explored is that of crowdsourcing ethics review [38], also called IRB 2.0. A code of conduct for data sharing in biocitizen project is also warranted [39], whereas concerns over security and privacy [40] are something that self-tracking individuals have so far ignored altogether.

## Discussion

The self-tracking movement has been slow to pick up with microbiome research, except for some individuals interested in the restoration of their ancestral gut microbiome [41], and all those trying fecal transplants at home without medical supervision [42]. As far as we know, Larry Smarr’s self-experimentation was the first published study that has looked at the relationship between gut microbiome and Crohn’s disease [11]. In a more recent self-tracking study, two researchers from Harvard and MIT have been using themselves as research subjects in a yearlong monitoring project of their gut (and oral) microbiome on a daily timescale [43]. Obviously, any given individual interested in replicating such experiments must have access to the technology itself or the funds necessary to purchase the technology or testing service, which is not easy if you are not already a scientist. However, looking at the range of companies currently offering to sequence your genome or microbiome (e.g., *American Gut Project*, *uBiome*, *my.microbes*, *DIYgenomics*, *Genomera*), the number of self-tracking projects making use of commercial kits to analyze microbiome is likely to grow in the years to come. As sequencing costs will go down, more and more people are going to perform self-experiments, and new citizen science projects conducted in collaboration with academic labs will gain in popularity. The accumulation of observations for single individuals may then provide insights about the dynamics of the microbiome in groups of individuals. Surely, this type of collaborative research provides an interesting and promising complementary approach to top-down, hypothesis-driven, and researcher-led research.

## Conclusion

Self-trackers are a special breed of self-experimenters, following in the footsteps of many others in the history of medicine [44]. They can perform experiments that clinical trials would never do, for lack of time, money, or interest. They can do so, more rapidly, extensively, and repeatedly. Despite the controversy about non-medically trained people taking full responsibility of their own bodies and making behavioral changes to achieve personal goals, this will not stop [11]. This trend is here is to stay and unlikely to be reversed. Instead of rejecting it on scientific grounds, we should address it by developing standard protocols for the framing of participant-led research involving self-tracking. Instead of refusing it on ethical basis, we should think about novel ways of assessing informed consent, anonymity, and transparency. A growing number of concerned individuals are demanding for socially robust citizen science [45], and self-trackers are a political force at the forefront of this movement. For sure, self-trackers have numbers on their side. By combining (*n* = 1) + (*n* = 1) + (*n* = 1) at infinitum, it is extremely likely that one of those *n* will make an important discovery. Namely, the first microbiome cocktail for treating *Clostridium difficile* infections was developed based on a study involving *n* = 2 subjects [46]. What’s the next breakthrough going to be? A cure for Crohn’s disease, psoriasis, depression, or any other microbiome-related condition, the end of obesity, or a revolutionary treatment for cancer patients? How many self-trackers will contribute to such discoveries? Not unlike Barry Marshall who received a Nobel Prize in 2005 for discovering the cause of gastritis by ingesting *Helicobacter pylori*, Jessica Richman, cofounder of *uBiome*, recently asked at a conference [47] whether a citizen scientist could win a Nobel Prize? Only time will tell.
